# Progress in Surgery

**Published:** 1895-08-24

**Authors:** 


### Progress in Surgery.

### SURGERY OF THE SPINE AND CORD.

**Injuries.**—Until our means of accurately diagnosing the precise situation and degree of spinal injuries are more perfect, it will be impossible to lay down more than general rules as to the appropriate treatment in cases of this class. One of the chief difficulties in the way of accurate diagnosis is the want of uniformity in the symptomatology of cases presenting the same pathological lesion. A case reported by Mansell Moullin[1] illustrates some of these difficulties. After falling into the hold of a ship, doubled up, the patient, while presenting none of the ordinary physical signs of fracture or dislocation of the spine, lost power completely, and sensation to a less extent, in the lower extremities. The sensory symptoms varied from time to time in degree, and various unusual and aberrant reflex phenomena were presented, notably reflex jerkings of the limbs, which became so severe as to necessitate the use of restraining apparatus. The vesical and rectal reflexes were also interfered with temporarily and in an irregular manner. The author believes that at worst the spinal lesion was a tearing of its ligaments; and that the nervous symptoms were due to a bruising of the cord, which was nipped at the moment of flexion, at a point between the last dorsal and first lumbar vertebræ. The case also throws some light on the vexed question of operation in such injuries. Clinically the case seemed a favourable one for surgical interference, but pathologically it was obviously quite inappropriate.

In discussing the *treatment* in cases of severe spinal injury Thorburn[2] indicates four principles on which it may be carried out: (1) Expectancy; (2) reduction of displacements; (3) laminectomy; (4) fixation by plaster of Paris or other support.

He mentions a case of his own treated *expectantly* in support of his statement that life may occasionally be indefinitely prolonged, even in cases which appear to be hopeless. The woman five and a half years ago sustained a fracture dislocation of the third dorsal vertebra—resulting in total paralysis and anæsthesia below that level, and there has been no material alteration in her symptoms since. Ringnell's[3] case also illustrates the effects of the expectant line of treatment. A young farmer dived into shallow water, and striking the bottom became temporarily unconscious. He could move no part of his body except his head, and to a very slight extent his arm, and he presented all the other signs and symptoms of a fracture with pressure in the middle cervical region. After a long tedious illness, complicated with cystitis and bedsores, he gradually began to mend, and in ten months was discharged from hospital with a marked enlargement in the region of the fourth cervical vertebra, but otherwise well. The treatment adopted was fixation of the head, the administration of potassium iodide from the sixth week onward, and later the use of massage and electricity.

With regard to treatment by *reduction*, Thorburn[2] states that unilateral dislocations are most favourable for this method, mainly because of the minor degree of displacement present, and the correspondingly slight damage to the cord. Bilateral luxations are reduced with much greater difficulty, and the displacement is readily reproduced, and in addition the cord is usually so seriously damaged that even after reduction marked improvement in the symptoms fails to take place. The prognosis is distinctly better when the lesion is low enough to involve the cauda equina, in which case recovery sometimes follows. Armstrong[4] reports a case of dislocation of the eighth or ninth dorsal vertebra, the result of forcible bending forward of the body, in which under an anæsthetic he and two assistants forcibly reduced the displacement, and the patient recovered without the development of any bad symptoms.

**Laminectomy.**—Surgical attention is at present more directed towards giving relief by operative measures than by less active means. Thorburn[2] does not lend the weight of his authority to this tendency. He says, "save in certain special conditions I do not believe that laminectomy is likely to be of service in injuries of the spine and spinal cord." Goldshreider[5] also takes a somewhat gloomy view of the value of operations for injury, and deprecates too hasty interference.

By some it has been stated that there are inherent dangers in operating upon the spinal cord, but Chipault, in his valuable work on the surgery of the nervous system,[6] denies this, and holds that operations on the cord are, *cæteris paribus*, less dangerous than operations on the encephalon. As might be expected, there is a very general concurrence of opinion that laminectomy is much more hopeful in injuries involving the cauda equina, than in lesions situated high enough to implicate the cord. Asepsis is of course a *sine quâ non*.

H. A. Burrell,[7] from a consideration of 168 cases of fracture of the spine, now inclines to operative treatment in preference to what he so long advocated—suspension, with forcible reduction and retention by plaster of Paris. Statistically he shows that there are 11 per cent. more recoveries by operation than by what he calls the "do-nothing treatment." He strongly insists on the operation being done early—the only contra-indication being shock.

Armison[8] also insists on early operation. He draws attention to the fact that if pulsation of the cord is observed at the operation it indicates a good prognosis. He does not advise opening the theca, and he has found that although much collapsed just after the operation the patients quickly rally. Riggs[9] reports two successful cases and supports early operation, with reduction by guarded but forced manipulation applied directly to the vertebræ themselves. Pyle[10] gives two cases, one successful and the other fatal. A case recorded by Sharples[11] goes to show that even when an operation is performed at once, and to all appearance the cause of the pressure symptoms is removed, success is by no means assured. The patient was crushed, and became paralysed below the waist. An operation enabled the fragments of the fractured eighth dorsal vertebra to be removed and the cord cleared, but no improvement followed. While favouring operation in these cases, Dundore[12] admits that the results are very uncertain, as is illustrated by three cases reported by him. One was operated on five months after injury, and the patient recovered. The other two, operated on respectively four months and twenty-four hours after injury, died unrelieved. Biddle,[13] without much show of reason, advises more frequent operation, as much for diagnostic as for curative purposes.

Salzer's case[14] is interesting chiefly on account of the manner in which the patient sustained his injury and the sequence of the symptoms. A house-painter, while at work, fell from a high building, and in his descent grasped at a projecting ledge, and managed to retain his hold till rescued. Beyond a pain in his back he complained of nothing at first, and continued his work for some time. Gradually, however, he lost power in his legs, and the pain increased. Three months' treatment by rest, extension, massage, and electricity failed to benefit him, and an abscess formed in the region of the eighth dorsal vertebra, which was drained. At a later date laminectomy was performed and disclosed a dislocation between the eleventh and twelfth dorsal vertebræ, which had caused compression and flattening of the cord. The patient was ultimately able to return to light work wearing a spinal support.

As an example of *gunshot injury* of the cord trea'ed by operation the case of Berezkine[15] may be cited. A young man shot himself in the precordial region, the missile lodging in the body of the eleventh dorsal vertebra. Laminectomy of the three last dorsal and first lumbar vertebræ enabled it to be removed, and the paraplegia, retention of urine and fæces, and anæsthesia which it had occasioned rapidly disappeared.

**Fixation** of the spine by jackets according to Thornton[2] is only useful as an adjunct to laminectomy, reduction, or spontaneous recovery, and is only to be employed during the period of convalescence, while parts are becoming consolidated.

**Spinal Caries—Pott's Disease.** — As in traumatic lesions of the spinal column and cord, so also in Pott's disease, an accurate diagnosis as to the seat and stage of the mischief is the only key to rational and successful treatment. In discussing the *morbid anatomy* of this condition from the standpoint of the neurologist, Burr[16] defines Pott's disease as "an osteitis, usually tuberculous, of one or more vertebræ, resulting in necrosis or caries." The changes in the cord, he holds, are not due to tuberculous processes taking place in its substance, but to the mechanical effects of pressure from inflammatory effusions, and possibly the irritant effect of retained lymph, the result of a passive œdema of the nervous tissues. This may lead on to sclerosis of the cord.

In papers dealing specially with the *treatment* of spinal caries, Noble-Smith[17] and Phelps[18] reiterate various points of importance, the former with regard to the appropriate treatment at different stages of the disease, the latter regarding practical points of detail in the application of plaster and other jackets.

**Laminectomy in Spinal Caries.**—The operation of laminectomy for the relief of paraplegia and other pressure effects of caries is gradually taking a forward place in the surgical treatment of this condition. Parkin[19] from a consideration of six cases of paraplegia strongly supports the operative means of treatment, on the ground that the paralytic symptoms are usually at once and permanently relieved; that the deformity can be much diminished; that direct local treatment can be applied to the focal mischief; and that the operation is not essentially a dangerous one. He has little faith in extension and counter-extension for paraplegia. Goldscheider,[20] however, takes a less hopeful view of the operation.

Successful cases have recently been recorded by Noble Smith,[21] A. Gray,[22] and F. C. Scheefer,[23] but the last writer's case was reported only four days after operation. Wyeth[24] reporting six cases, offers some remarks on the technique of the operation, and concludes that the operation is not difficult or dangerous, and that paraplegia justifies early exploration, especially in the presence of evidence of degenerative changes.

M. Fontan[25] describes a new operation for reaching the bodies of the vertebræ; he makes an incision one centimetre from the middle line, splits the common mass of muscles in order to come upon the apophyseal tubercle; then outside this he plunges in his finger in order to recognise the costal apophysis which lies at a deeper level. Then with a chisel he cuts this at its base, seizes it, and excises it completely. From here he follows the vertebral column from behind forwards with the raspatory, keeping close to the bone under the periosteum, until he reaches the seat of the caries.

Discussing the question of the *treatment of iliac abscesses* of spinal origin, James K. Young[26] advocates an antiseptic cutting operation in preference to such measures as aspiration, injection of fluids to promote absorption or expectancy. He dissects down on the abscess, and after opening it and flushing it out with boiled water or boracic lotion, he makes a counter opening above the sacro-iliac joint, and passes a drainage tube through. Iodoform emulsion is thrown in, and the wound packed with iodoform gauze. Taylor,[26] in addition to this, curettes the interior of the abscess.

**Sciatica as an Early Symptom in Pott's Disease.**—Farrar[27] draws attention to the importance of careful discrimination in the diagnosis of sciatica, which he states to be often the first and for many months the only thing complained of in cases of disease of the lumbar vertebræ, even with iliac abscess. Until such gross causes of sciatic pain have been eliminated, it is unjustifiable to set down the condition as one of neuralgia. As pointed out by Eskridge,[28] such errors in diagnosis are only likely to occur when the sciatic pain is unilateral, bilateral sciatica being exceedingly rare, and at once suggesting a spinal cause. It is also to be borne in mind that in neuritis due to pressure, the nerve below the point of pressure may be the seat of pain; it is not tender until sufficient time has elapsed to affect the peripheral portion of the nerve, and the pain is greatest distally. Sciatica may also be closely simulated by certain tumours of the spinal column, *e.g.*, Bennet's case[29] of a woman, 46, who suffered from double sciatica, which ordinary treatment failed to relieve. It was discovered that the pain was due to a malignant tumour of the spine, secondary to a mammary growth.

[1] Lancet, Nov. 3, 1894. [2] B. M. J., Oct. 27, 1894. [3] N. Y. Med. Rec., Mar. 9, 1895. [4] Montreal Med. Jour., Mar., 1894. [5] Deut. Med. Woch., July 19, 26, 1859. [6] Chirurgie Operatoire au Sys. Nerv., 1895. [7] B. M. J., Oct. 27, 1894. [8] Ann. Surg., May, 1895. [9] Ann. Surg., June, 1894. [10] Ann. Surg., June, 1894. [11] Philadelphia Med. News, 1894. [12] Med. News, Nov. 24, 1894. [13] Lehigh Valley Med. Mag., Ap., 1895. [14] Weekblad von het Nederlandsch, 1894. [15] La Tribune Médicale, June 7, 1894. [16] Med News, Nov., 1893. [17] Med. Press and Circular, May, 1894. [18] Jour. Amer. Med. Assoc., 1894. [19] Internat. Jour. of Surg., Jan., 1895; B. M. J., Sep., 1894. [20] Deut Med. Woch, July, 1894. [21] B. M. J., Dec., 1894. [22] B. M. J., Ap., 1895. [23] Internat. Med. Mag., 1894. [24] Am. Surg., Aug., 1894. [25] Revue de Chirurgie, 1894. [26] N. Y. Med. Jour., Dec., 1894. [27] Quart. Med. Jour., Oct., 1894. [28] N. Y. Med. Jour., Nov., 1894. [29] Clin. Jour., Oct., 1894.

